# Relationships between mammographic density, tissue microvessel density, and breast biopsy diagnosis

**DOI:** 10.1186/s13058-016-0746-9

**Published:** 2016-08-23

**Authors:** Ashley S. Felix, Petra Lenz, Ruth M. Pfeiffer, Stephen M. Hewitt, Jennifer Morris, Deesha A. Patel, Berta Geller, Pamela M. Vacek, Donald L. Weaver, Rachael E. Chicoine, John Shepherd, Amir Pasha Mahmoudzadeh, Jeff Wang, Bo Fan, Serghei Malkov, Sally D. Herschorn, Jason M. Johnson, Renata L. Cora, Louise A. Brinton, Mark E. Sherman, Gretchen L. Gierach

**Affiliations:** 1 Division of Cancer Epidemiology and Genetics, National Cancer Institute, National Institutes of Health, Bethesda, MD USA; 2 Cancer Prevention Fellowship Program, Division of Cancer Prevention, National Cancer Institute, National Institutes of Health, Bethesda, MD USA; 3 Present address: Division of Epidemiology, The Ohio State University College of Public Health, 1841 Neil Avenue, 300C Cunz Hall, Columbus, OH 43210 USA; 4 Clinical Research Directorate/Clinical Monitoring Research Program, Leidos Biomedical Research, Inc., Frederick National Laboratory for Cancer Research, Frederick, MD USA; 5 Laboratory of Pathology, Center for Cancer Research, National Cancer Institute, National Institutes of Health, Bethesda, MD USA; 6 Department of Family Medicine, University of Vermont, Burlington, VT USA; 7 Department of Pathology, University of Vermont, Burlington, VT USA; 8 Office of Health Promotion Research, University of Vermont, Burlington, VT USA; 9 University of California, San Francisco, CA USA; 10 Present address: Hokkaido University, Graduate School of Medicine, Sapporo, Japan; 11 Department of Radiology, University of Vermont, Burlington, VT USA; 12 Department of Diagnostic Radiology, Neuroradiology Section, MD Anderson Cancer Center, Houston, TX USA; 13 Division of Cancer Prevention, National Cancer Institute, National Institutes of Health, Bethesda, MD USA

**Keywords:** Mammographic density, Breast neoplasms, Angiogenesis, Pathology, Lesional density

## Abstract

**Background:**

Women with high levels of mammographic density (MD) have a four- to six-fold increased risk of developing breast cancer; however, most neither have a prevalent tumor nor will they develop one. Magnetic resonance imaging (MRI) studies suggest that background parenchymal enhancement, an indicator of vascularity, is related to increased breast cancer risk. Correlations of microvessel density (MVD) in tissue, MD and biopsy diagnosis have not been defined, and we investigated these relationships among 218 women referred for biopsy.

**Methods:**

MVD was determined by counting CD31-positive vessels in whole sections of breast biopsies in three representative areas; average MVD was transformed to approximate normality. Using digital mammograms, we quantified MD volume with single X-ray absorptiometry. We used linear regression to evaluate associations between MVD and MD adjusted for age and body mass index (BMI) overall, and stratified by biopsy diagnosis: cases (in situ or invasive cancer, n = 44) versus non-cases (non-proliferative or proliferative benign breast disease, n = 174). Logistic regression adjusted for age, BMI, and MD was used to calculate odds ratios (ORs) and 95 % confidence intervals (CIs) for associations between MVD and biopsy diagnosis. We also assessed whether the MVD-breast cancer association varied by MD.

**Results:**

MVD and MD were not consistently associated. Higher MVD was significantly associated with higher odds of in situ/invasive disease (OR_Adjusted_ = 1.69, 95 % CI = 1.17–2.44). MVD-breast cancer associations were strongest among women with greater non-dense volume.

**Conclusions:**

Increased MVD in tissues is associated with breast cancer, independently of MD, consistent with MRI findings suggestive of its possible value as a radiological cancer biomarker.

**Electronic supplementary material:**

The online version of this article (doi:10.1186/s13058-016-0746-9) contains supplementary material, which is available to authorized users.

## Background

Mammographic density (MD), a radiographic reflection of the proportion of fibroglandular tissue in the breast, is one of the strongest risk factors for the development of ductal carcinoma in situ (DCIS) and invasive breast cancer [1, 2]. Nonetheless, most women with high MD have neither a prevalent tumor nor will they develop one during follow-up. Accordingly, identifying additional risk markers that are independent of MD may improve risk prediction.

Multiple breast imaging techniques [3], including breast magnetic resonance imaging (MRI), can be used to evaluate breast density, and most yield similar results with respect to breast cancer risk. In addition, recent data suggest that MRI performed with contrast provides a measure of background parenchymal enhancement (BPE), which reflects vascularity and vessel permeability [4] and has been associated with breast cancer odds in cross-sectional studies [5, 6]. Morphologically, the distribution, shape and size of radiologic patterns of dense tissue align well with areas of non-fatty tissue on gross examination of surgical pathology specimens; however, microscopically, areas of dense tissue appear highly variable, ranging from hypocellular dense collagen to hypercellular regions of invasive carcinoma. We hypothesized that vascularity is correlated with cellularity, suggesting that measurement of the former might prove useful in distinguishing dense tissues harboring neoplastic lesions from benign fibrosis. Accordingly, to assess this hypothesis, we examined relationships between microvessel density (MVD), a commonly used histologic measure of tumor angiogenesis, with MD measures and concurrent breast biopsy diagnoses.

## Methods

### Study population

This study included 465 women aged 40–65 years undergoing image-guided breast biopsies at the University of Vermont College of Medicine and the University of Vermont Medical Center that participated in the National Cancer Institute (NCI) Breast Radiology Evaluation and Study of Tissues (BREAST) Stamp Project from October 2007 to June 2010, as previously described [7]. Briefly, this study focused on patients referred for a diagnostic image-guided breast biopsy. At the time of mammogram, study participants completed a standard health history questionnaire which assessed known breast cancer risk factors. When a breast imaging study was considered abnormal, indicating the need for a biopsy, women were contacted by the study coordinator and screened to determine eligibility, obtain verbal consent, and administer an approximately 20-minute telephone interview to collect additional health information, including history of exogenous hormone use. Ninety percent of study participants underwent biopsy within 10 days of completing this interview. Eligible women were those without a history of breast cancer or treatment, who had not undergone breast surgery within the preceding year, did not have breast implants, were not taking breast cancer chemoprevention and were scheduled to have an image-guided breast biopsy. On the day of the breast biopsy, a research coordinator measured participants’ height and weight.

### Mammographic density assessment

Mammograms were acquired on one of six full-field digital mammography systems. Raw images were encrypted and transferred to the University of California at San Francisco for quantitative volume and area density assessment. This analysis was restricted to pre-biopsy views [craniocaudal (96 %) or mediolateral oblique (4 %)] of the ipsilateral breast. For three women who underwent bilateral breast biopsies, the breast with the most severe diagnosis was selected for analysis. If more than one mammogram was available, the mammogram taken closest in time prior to the breast biopsy date was selected.

Mammographic density (global and lesional) was quantified as an absolute tissue volume (cm^3^) and percent tissue volume using single X-ray absorptiometry (SXA), as described previously [8, 9]. An SXA breast density phantom was affixed to the top of the compression paddle and included in the X-ray field during mammography examinations. Mammographic grayscale values were compared to the values of the SXA phantom with a known fibroglandular tissue volume (FGV) composition and thickness. In this way, volumetric measures were achieved using a planar image. Previous estimates of reproducibility for the SXA test phantoms demonstrated a repeatability standard deviation of 2 %, with a ±2 % accuracy for the entire thickness and density ranges [8]. Area-based mammographic density measures were also available as previously reported in this study population [7]. As volume and area measures were highly correlated [7], we limit our presentation of results to volumetric measures.

To compute localized density measures of the biopsied lesion, two radiologists (SDH, JMJ) recorded the biopsy location and radius of the biopsy target on the pre-biopsy standard digital mammogram (i.e., Digital Imaging and Communications in Medicine format). Absolute lesional volume (cm^3^) and percent lesional volume were estimated using SXA within the biopsy target, centered at the biopsy site [9]. A repeat set of 25 images was assessed for reliability. The intraclass correlation coefficients (ICCs) for percent lesional volume, absolute lesional volume, and total lesional volume were 0.99, 0.50, and 0.44 respectively, indicating fair to excellent reproducibility. Distributions of density measures were examined and images with extreme values were reviewed visually for validation. The American College of Radiology’s Breast Imaging Reporting and Data System (BI-RADS, Fourth Edition) breast density assessment (reported on the same images used for quantitative analysis) was analyzed as (I) almost entirely fat; (II) scattered fibroglandular densities; (III) heterogeneously dense; and (IV) extremely dense [10].

### Pathologic diagnosis assessment

Pathology reports from the breast biopsy and surgical excision were reviewed to assign final pathologic diagnoses. We classified the following diagnoses as non-cases: non-proliferative benign breast disease, proliferative (ductal hyperplasia; sclerosing adenosis), and proliferative with atypia (atypical ductal or lobular hyperplasia) (n = 174). Cases included diagnoses of ductal or lobular carcinoma in situ (n = 36) and invasive cancer (n = 8).

As part of the clinical workup, tumor blocks from in situ and invasive cancer were sectioned and stained for estrogen receptor (ER) and progesterone receptor (PR). We obtained these slides and one observer (RLC) rescored all sections microscopically. The percentage of stained cells (range: 0–100 %) and intensity (0 = negative, 1 = weak, 2 = moderate, 3 = strong) were recorded and an overall score was calculated as the product of these components (range: 0–300). We dichotomized expression as negative (<10) or positive (≥10). When information on these stains was missing, we obtained tumor blocks and performed immunohistochemistry according to routine protocols, also scored by the same microscopist (RLC).

### CD31 immunohistochemical staining and MVD assessment

Paraffin-embedded whole section slides were deparaffinized and antigen retrieval performed with citrate buffer pH6 using a pressure cooker. Endogenous activity was blocked with 3 % peroxidase and primary antibody CD31 (Clone JC70A, DAKO, Glostrup, Denmark) was applied for 2 hours at room temperature. Subsequently, antibody was detected using DAKO Env + labeling system and visualized with 3,3′-diaminobenzadine (DAB) for 20 min. After rinsing, slides were counterstained with modified Harris hematoxylin, dehydrated with graded alcohol, cleared with xylene and a coverslip applied.

**Fig. 1:**
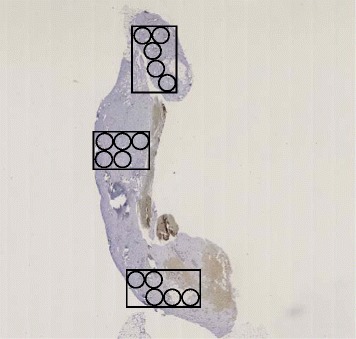
Assessment of microvessel density (MVD) in CD31-stained whole section slides. Three random regions from the top, central, and bottom portions of the whole section slide were selected. Within each region, five adjacent, non-overlapping × 400 high-power fields were assessed for the number of CD31-staining microvessels

We performed a pilot study including 20 participants to determine the within-woman variability of MVD on whole section slides, based on counting vessels within three regions, each consisting of ten × 400 high-power fields, randomly selected from the (1) top, (2) center, and (3) bottom portions of the slide. When multiple tissue fragments existed, one randomly selected region from each of the three largest fragments was selected for MVD counting. Coefficient of variations (CVs) and ICCs were similar based on analyzing 30 or 15 fields; accordingly, in the full study, 15 MVD scores per slide were assessed by a pathologist (PL) masked to biopsy diagnosis (see Fig. 1). We analyzed the average MVD score per woman.

A masked independent review of 20 randomly selected slides after study completion by two pathologists (PL, MES) demonstrated an intra-rater agreement (ICC) of 0.88 and inter-rater agreement (ICC) of 0.82.

### Analytic population

Of 465 participants enrolled, 12 were not subsequently biopsied and two went straight to surgery without a biopsy and were excluded. We excluded women who underwent ultrasound-guided core (n = 225) and MR-guided vacuum-assisted biopsies (n = 1). Ultrasound-guided biopsies were generally used to sample mass lesions, providing less non-lesional tissue for MVD analysis than vacuum-assisted breast biopsies, and were therefore excluded. We further excluded participants with invalid SXA volumetric MD measurements (n = 5) and insufficient tumor tissue on slide (n = 2), resulting in a final sample of 218 women.

### Statistical analysis

Relationships between baseline characteristics according to final pathologic diagnosis (in situ and invasive cancer versus benign diagnoses) were examined using chi-square or Fisher’s exact tests for categorical variables and Wilcoxon rank sum tests for continuous variables. Spearman rank correlation coefficients were computed to estimate correlations of MVD with age at biopsy, body mass index (BMI), and MD measures overall and by final pathologic diagnosis. Based on a Box-Cox transformation analysis, we used a power of 0.2 to improve normality of the average MVD score. Age- and BMI-adjusted linear regression models were used to examine relationships between MVD with participant characteristics and MD measures overall and by case status. We tested for differences by case status by including a multiplicative term between participant characteristics and MD measures with a binary indicator for case status and evaluating the Wald test *p* value. Age at biopsy (39–44, 45–49, 50–54, 55–59, 60–65 years) and BMI (<25, 25– < 30, 30+ kg/m^2^) were coded as categorical variables. Other breast cancer risk factors were not significantly associated with MVD, and were therefore not included as potential confounders. We also examined the relationship between MVD and MD measures stratified by menopausal status based on previous findings demonstrating a decline in BPE after menopause in some women [11].

Logistic regression models were used to estimate odds ratios (ORs) and 95 % confidence intervals (CIs) for associations between MVD (the average of MVD within a woman was standardized by one standard deviation) and final pathologic diagnosis (benign versus in situ/invasive) adjusted for age at biopsy and BMI. Subsequent models were adjusted for individual MD measures. We also evaluated whether a “gradient” of MVD and lesion severity existed using polytomous logistic regression with the following outcome categories: (1) benign, carcinoma in situ (ductal or lobular), and invasive cancer and, in a second model, (2) benign, proliferative, proliferative with atypia, carcinoma in situ (ductal or lobular) and invasive cancer. We also examined the association between MVD and final pathologic diagnosis stratified by MD. In these analyses, each MD measure was dichotomized at the median based on the distribution among the non-cases. We formally tested whether the ORs in each MD stratum were significantly different by including a multiplicative term between the dichotomous MD variable and MVD and evaluating the Wald test *p* value. We used SAS (version 9.3, SAS Institute, Inc., Cary, NC, USA) software for all analyses and two-sided *p* values less than 0.05 were considered statistically significant.

## Results

### Participant characteristics

Among the non-cases, 50 had benign diagnoses, 99 had proliferative diagnoses, and 25 had proliferative disease with atypia (Table 1). Cases reported past menopausal hormone therapy use (40.9 % vs. 20.1 %) and current use of oral contraceptives (9.1 % vs. 1.7 %) more frequently than non-cases. Global volumetric density did not significantly differ between cases and non-cases; however, lesional non-dense volumes were significantly higher in cases compared with non-cases. In univariate analysis, MVD in cases and non-cases was not significantly different (2.5 vs. 2.2, Kruskal-Wallis *p* value = 0.13).

**Table 1:** Selected characteristics of women referred to an image-guided vacuum-assisted breast biopsy in The BREAST Stamp Project by breast biopsy diagnosis (n = 218)

Characteristics	Non-cases (n = 174)		Cases (n = 44)		*p* ^a^
Median age at biopsy (range)	52 (40–65)		52 (40–64)		0.25
	n	%^b^	n	%^b^	
Age at biopsy (years)					0.11
39 to 44	25	14.4	6	13.6	
45 to 49	46	26.4	5	11.4	
50 to 54	51	29.3	13	29.5	
55 to 59	26	14.9	13	29.5	
60 to 65	26	14.9	7	15.9	
Race					0.99
White	159	91.4	41	93.2	
Non-white	15	8.6	3	6.8	
Education					0.53
< High school or high school graduate	26	15.1	5	11.4	
College/graduate school degree	146	84.9	39	88.6	
Cigarette smoking (≥100)					0.24
Never	83	48.5	17	38.6	
Ever	88	51.5	27	61.4	
BMI (kg/m^2^)					0.49
< 25	92	52.9	24	54.5	
25–<30	45	25.9	8	18.2	
≥ 30	37	21.3	12	27.3	
Age at menarche					0.79
≤ 12	65	37.8	18	40.9	
13	63	36.6	17	38.6	
≥ 14	44	25.6	9	20.5	
Oral contraceptives					**0.02**
Never	21	12.2	8	18.2	
Former	148	86.1	32	72.7	
Current	3	1.7	4	9.1	
Parity					0.17
Nulliparous	46	26.4	6	13.6	
1	23	13.2	10	22.7	
2	68	39.1	20	45.5	
≥ 3	37	21.3	8	18.2	
Age at first birth (years)					0.62
< 30	91	52.6	25	56.8	
Nulliparous or ≥ 30	82	47.4	19	43.2	
Menopausal status					0.24
Premenopausal	100	57.5	21	47.7	
Postmenopausal	74	42.5	23	52.3	
Age at menopause (years)					0.59
Premenopausal	100	57.5	21	47.7	
< 45	12	6.9	4	9.1	
45–49	17	9.8	3	6.8	
≥ 50	32	18.4	12	27.3	
Postmenopausal, age unknown	13	7.5	4	9.1	
Menopausal hormone therapy					**0.04**
Never	121	69.5	23	52.3	
Former	35	20.1	18	40.9	
Current	10	5.7	2	4.5	
Missing/unknown	8	4.6	1	2.3	
Family history of breast cancer					0.83
No	131	75.7	34	77.3	
Yes	42	24.3	10	22.7	
Lump at the time of mammography					0.47
No	159	91.4	43	97.7	
Yes	8	4.6	1	2.3	
Missing/unknown	7	4.0	0	0.0	
Reason for mammography					0.91
Screening	156	89.7	41	93.2	
Short-interval follow-up	10	5.7	2	4.5	
Evaluation of breast problem	8	4.6	1	2.3	
BI-RADS breast density					0.74
I (entirely fat)	20	11.5	3	6.8	
II (scattered densities)	78	44.8	20	45.5	
III (heterogeneously dense)	51	29.3	17	38.6	
IV (extremely dense)	14	8.0	2	4.5	
Missing/unknown	11	6.3	2	4.5	
Final BI-RADS mammography assessment					**0.03**
3 (probably benign finding)	3	1.7	1	2.3	
4 (suspicious abnormality)	169	97.1	39	88.6	
5 (highly suggestive of malignancy)	1	0.6	3	6.8	
Biopsy laterality					0.64
Left	82	47.1	24	54.5	
Right	89	51.1	20	45.5	
Bilateral	3	1.7	0	0.0	
Pathologic diagnosis^c^					
Benign	50	28.7	--	--	
Proliferative	99	56.9	--	--	
Proliferative with atypia	25	14.4	--	--	
In situ	--	--	36	81.8	
Invasive carcinoma	--	--	8	18.2	
Lesion size					0.24
< 1 cm	93	54.1	19	44.2	
≥ 1 cm	79	45.9	24	55.8	
Volumetric mammographic density measures	Median	Range	Median	Range	
Global					
% density (volume)	36.0	0.6–99.3	35.2	10.0–97.3	0.59
Dense volume (cm^3^)	178.6	6.7–497.8	177.0	80.3–683.5	0.70
Non-dense volume (cm^3^)	324.7	1.6–1977.0	433.0	2.2–1291.5	0.31
Lesional					
% density (volume)	42.4	0.0–100.0	44.5	6.1–100.0	0.86
Dense volume (cm^3^)	1.4	0.0–26.8	1.7	0.2–16.9	0.08
Non-dense volume (cm^3^)	1.4	0.0–26.3	2.8	0.0–37.1	**0.03**
MVD^d^	2.2	0.6–5.0	2.5	1.0–5.0	0.13

Variables with *P* values < 0.05 are presented in bold font

*BREAST* Breast Radiology Evaluation and Study of Tissues, *BMI* body mass index, *BI-RADS* Breast Imaging Reporting and Data System, *MVD* microvessel density

^a^Chi-square test was used for categorical variables; where 25 % of cells had counts less than 5 we used Fisher’s exact test. Wilcoxon rank sum test was used for continuous variables

^b^When more than 3 % of data were missing for a covariate, missing values were included as a separate category. Otherwise, missing data were excluded from column percentages and chi-square or Fisher’s exact test

^c^Benign: normal lobules or ducts defined as sclerotic/atrophied; non-proliferative fibrocystic change; discrete entities. Proliferative: ductal hyperplasia; sclerosing adenosis. Proliferative with atypia: atypical ductal or lobular hyperplasia

^d^The average of MVD within a woman was computed and standardized by one standard deviation

### Relationships between MVD with participant characteristics and MD measures

Among non-cases, MVD was inversely correlated with age at biopsy (rho = -0.33, *p* < 0.0001) and BMI (rho = -0.28, *p* < 0.0002) (data not tabled). In multivariable linear regression models adjusted for age and BMI, MVD was inversely associated with age at biopsy and BMI among non-cases (Table 2). Among cases, MVD was higher among women with three or more live births (*p* = 0.04). We did not observe significant associations between MVD and other risk factors.

**Table 2:** Age- and body mass index (BMI)-adjusted linear regression results for the association between participant characteristics and microvessel density (MVD), overall and by case status

	All women		Non-cases		Cases		
	N = 218		n = 174		n = 44		
Characteristics	β^a^	*p*	β^a^	*p*	β^a^	*p*	*P* heterogeneity^b^
Age (years)		0.009		0.01		0.46	0.63
45 to 49	-0.03		-0.03		0.01		
50 to 54	-0.07		-0.07		-0.07		
55 to 59	-0.08		-0.11		-0.03		
60 to 65	-0.10		-0.09		-0.12		
BMI (kg/m^2^)		0.09		0.02		0.87	0.24
25– < 30	-0.009		-0.01		0.02		
≥ 30	-0.05		-0.07		0.03		
Age at menarche (years)		0.53		0.77		0.49	0.68
13	0.02		0.01		0.05		
≥ 14	0.02		0.01		0.07		
Oral contraceptive use		0.81		0.68		0.62	0.44
Ever	-0.01		-0.01		0.03		
Parity		0.52		0.89		0.04	0.02
1	-0.01		-0.02		0.03		
2	-0.01		-0.01		-0.01		
≥ 3	0.02		-0.01		0.16		
Age at first birth (years)		0.33		0.29		0.98	0.83
Nulliparous or ≥ 30	0.02		0.02		0.00		
Menopausal status		0.14		0.43		0.25	0.99
Postmenopausal	-0.04		-0.02		-0.09		
Age at menopause (years)		0.08		0.11		0.68	0.92
< 45	-0.10		-0.09		-0.02		
45–49	-0.003		0.02		-0.11		
≥ 50	-0.06		-0.05		0.08		
Menopausal hormone therapy		0.73		0.66		0.52	0.75
Ever	0.01		-0.01		0.03		
Family history of breast cancer		0.76		0.87		0.76	0.92
Yes	-0.01		-0.003		-0.02		

Non-cases: non-proliferative benign breast disease, proliferative (ductal hyperplasia; sclerosing adenosis), proliferative with atypia (atypical ductal or lobular hyperplasia). Cases: ductal or lobular carcinoma in situ and invasive cancer.

^a^Based on linear regression with the Box-Cox-transformed average MVD (within a woman) as the outcomeand adjusted for age at biopsy (39–44, 45–49, 50–54, 55–59, 60–65 years) and BMI (<25, 25– < 30, 30+ kg/m^2^)

^b^ *P* heterogeneity based on a Wald test in the regression model corresponding to an interaction term between case status and the corresponding variable

Age- and BMI-adjusted linear regression models were used to evaluate associations between MVD and the MD measures (Table 3). MVD was not significantly associated with global or lesional percent dense volume measures. Among all women and the non-cases, MVD tended to be inversely associated with global measures of both absolute dense and non-dense volume. In particular, among all women, we observed statistically significant inverse associations between MVD and non-dense volume (β = -0.00007, *p* = 0.04). Among non-cases, MVD was inversely related to absolute dense volume (β = -0.0003, *p* = 0.008) and non-dense volume (β = -0.00008, *p* = 0.01). We observed positive associations of MVD with non-dense lesional volumes among all women and among cases (*p* ≤ 0.04). Finally, when the association between MVD and MD measures was stratified by menopausal status, we did not observe significant deviations from these patterns (data not tabled).

**Table 3:** Age- and body mass index (BMI)-adjusted linear regression results for the association between microvessel density (MVD) and mammographic density (MD) measures, overall and by case status

	All women		Non-cases		Cases		
	N = 218		n = 174		n = 44		
MD measure	β^a^	*p*	β^a^	*p*	β^a^	*p*	*P* heterogeneity^b^
Global							
% density (volume)	0.0004	0.35	0.0003	0.49	0.0005	0.70	0.67
Dense volume (cm^3^)	-0.0001	0.11	**-0.0003**	**0.008**	-0.00004	0.84	0.43
Non-dense volume (cm^3^)	**-0.00007**	**0.04**	**-0.00008**	**0.01**	0.00001	0.89	0.31
Lesional							
% dense volume (cm^3^)	0.0005	0.16	0.0006	0.16	0.0001	0.93	0.83
Dense volume (cm^3^)	0.004	0.08	0.002	0.37	0.01	0.15	0.22
Non-dense volume (cm^3^)	**0.004**	**0.03**	0.0003	0.90	**0.006**	**0.04**	0.09

Beta coefficients with *P* values < 0.05 are presented in bold font. Non-cases: non-proliferative benign breast disease, proliferative (ductal hyperplasia; sclerosing adenosis), proliferative with atypia (atypical ductal or lobular hyperplasia). Cases: ductal or lobular carcinoma in situ and invasive cancer

^a^Based on linear regression with the Box-Cox-transformed, standardized average MVD (within a woman) as the outcome and adjusted for age at biopsy (39–44, 45–49, 50–54, 55–59, 60–65 years) and BMI (<25, 25– < 30, 30+ kg/m^2^)

^b^ *P* heterogeneity based on a Wald test in the regression model corresponding to an interaction term between case status and the corresponding MD measure

### Associations between MVD and breast cancer odds

In logistic regression models adjusted for age and BMI, we observed that a one standard deviation increase in MVD was associated with a significant increased OR of carcinoma in situ*/*invasive disease compared with non-cases (OR = 1.69, 95 % CI = 1.17–2.44) (Table 4). Compared with non-cases, MVD was positively associated with carcinoma in situ (OR = 1.36, 95 % CI = 0.91–2.04) and invasive cancer (OR = 4.86, 95 % CI = 1.91–12.38) (Table 4). In models that further parsed the biopsy diagnoses as benign, proliferative, proliferative with atypia, carcinoma in situ, and invasive cancer, we observed a significant increase in the proportional odds of more invasive disease associated with higher MVD (OR = 1.66, 95 % CI = 1.27 - 2.16) (Table 4). These relationships persisted after further adjustment for MD measures.

**Table 4:** Logistic regression estimates for the association between microvessel density (MVD) and breast biopsy diagnosis

Breast biopsy diagnosis	N	OR (95 % CI)^a^	*P*
Non-case	174	1.00	0.005
Case	44	1.69 (1.17–2.44)	
Non-case	174	1.00	0.002
In situ carcinoma	36	1.36 (0.91–2.04)	
Invasive	8	4.86 (1.91–12.38)	
Benign	50	1.00	0.002
Proliferative	99	1.56 (1.04–2.33)	
Proliferative with atypia	25	1.47 (0.84–2.58)	
In situ carcinoma	36	1.89 (1.13–3.15)	
Invasive	8	6.81 (2.52–18.42)	
Ordinal odds ratio		1.66 (1.27–2.16)	0.002

The average of MVD within a woman was computed and standardized by one standard deviation. Non-cases: non-proliferative benign breast disease, proliferative (ductal hyperplasia; sclerosing adenosis), proliferative with atypia (atypical ductal or lobular hyperplasia). Cases: ductal or lobular carcinoma in situ and invasive cancer

^a^Adjusted for age at biopsy (39–44, 45–49, 50–54, 55–59, 60–65 years) and BMI (<25, 25– < 30, 30+ kg/m^2^)

To test our primary hypothesis of an interaction between MVD and MD, Table 5 presents associations between MVD and breast cancer odds stratified by MD measures. We observed increased breast cancer odds with increasing MVD among women classified as low (i.e., below the median) percent density, which was consistent across MD measures. For example, among women with low global or low lesional percent dense volume, higher MVD was associated with higher breast cancer odds (OR = 2.35, 95 % CI = 1.33–4.14) and (OR = 3.03, 95 % CI = 1.61–5.70), respectively. The effect estimates in the low percent density stratum significantly differed from the effects observed in the high (i.e., above the median) percent density stratum for lesional density (*p* heterogeneity = 0.03) but not global density (*p* heterogeneity = 0.11).

**Table 5:** Logistic regression estimates for the association between microvessel density (MVD) and breast biopsy diagnosis stratified by mammographic density (MD) measures

Density measure	Below median of mammographic density measure			Above median of mammographic density measure			
	Cases	Non-cases	OR (95 % CI)^a^	Cases	Non-cases	OR (95 % CI)^a^	*P* heterogeneity^b^
Volume	Effect of microvessel density (MVD)^c^ on breast cancer risk						
Global							
% density (volume)	23	87	**2.35 (1.33–4.14)**	21	87	1.23 (0.73–2.07)	0.11
Dense volume (cm^3^)	22	87	1.60 (0.84–3.05)	22	87	**1.78 (1.07–2.98)**	0.23
Non-dense volume (cm^3^)	21	87	1.14 (0.67–1.95)	23	87	**2.51 (1.41–4.46)**	0.08
Lesional							
% dense volume (cm^3^)	21	88	**3.03 (1.61–5.70)**	23	86	1.10 (0.69–1.79)	**0.03**
Dense volume (cm^3^)	19	88	1.25 (0.67–2.32)	25	86	**1.90 (1.15–3.13)**	0.61
Non-dense volume (cm^3^)	15	87	0.97 (0.50–1.90)	29	87	**2.13 (1.30–3.48)**	0.06

Each MD measure was dichotomized at the median based on the distribution among non-cases. ORs with *P* values < 0.05 are presented in bold font. Non-cases: non-proliferative benign breast disease, proliferative (ductal hyperplasia; sclerosing adenosis), proliferative with atypia (atypical ductal or lobular hyperplasia). Cases: ductal or lobular carcinoma in situ and invasive cancer

^a^Adjusted for age at biopsy (39–44, 45–49, 50–54, 55–59, 60–65 years) and BMI (<25, 25– < 30, 30+ kg/m^2^)

^b^ *P* heterogeneity based on a Wald test in the regression model corresponding to an interaction term between the dichotomous MD measure and MVD

^c^The average of MVD within a woman was computed and standardized by one standard deviation

Among women categorized as high (i.e., above the median) absolute dense volume, higher MVD was significantly associated with increased breast cancer odds. For example, among women with high global absolute dense volume, higher MVD was associated with a 1.78-fold increase in breast cancer odds (95 % CI = 1.07–2.98); however, this was not significantly different than the odds observed among women in the low global absolute dense volume category (OR = 1.60, 95 % CI = 0.84–3.05, *p* heterogeneity = 0.23). A similar pattern was noted for lesional absolute dense volume.

Regarding the non-dense volume measures, the association between MVD and breast cancer differed between women with lower versus higher amounts of non-dense volumes, such that higher breast cancer odds were observed among women with greater amounts of non-dense tissues. For example, higher MVD was associated with a 2.51 (95 % CI = 1.41–4.46) times higher odds of breast cancer among women in the high absolute non-dense volume stratum compared with a 1.14 (95 % CI = 0.67–1.95) times higher breast cancer odds among women in the low absolute non-dense volume stratum (*p* heterogeneity = 0.08). A similar pattern was noted for lesional non-dense volume.

Lastly, relationships between tumor characteristics and MVD were evaluated among the 44 cases (Additional file 1: Table S1). We did not observe significant differences by grade, histology, or hormone receptor positivity; however, this exploratory analysis was based on small numbers.

## Discussion

Our results demonstrate that women with diagnostic vacuum-assisted breast biopsies and a pathology finding of in situ or invasive breast cancer are more likely than women with benign lesions to have higher tissue MVD. MVD was not associated with MD, although the relationship of higher MVD and in situ/invasive breast cancer was higher among women with increased total breast adipose content. These findings are consistent with data suggesting that women whose MRIs show higher BPE are more likely to have a prevalent breast cancer, and support further research on parenchymal patterns in breast imaging as markers of breast cancer risk.

MRI-assessed BPE is directly related to vascular supply and vessel permeability [4], which enables the detection of breast cancer secondary to increased blood flow and leaking of contrast from abnormally permeable cancer-associated vessels. Our findings are consistent with two retrospective breast MRI studies, in which higher levels of MRI-assessed BPE were associated with breast cancer independent of MRI-assessed fibroglandular tissue (i.e., density). In the study by King and colleagues [5], among 1275 women who underwent breast MRI screening, 39 women had a prevalent breast cancer. Increased BPE was strongly associated with elevated breast cancer odds after adjustment for fibroglandular tissue [5]. In the second MRI study to evaluate BPE level and breast cancer odds, 23 women with a breast cancer diagnosed a median of 2 years after the index MRI and 23 age- and *BRCA1/2* mutation status-matched women who did not develop breast cancer were included. Compared with minimal BPE, breast cancer risk was nine times higher (95 % CI = 1.1–71.0) among those with mild, moderate, or marked BPE [6]. This latter study suggests that MRI-assessed BPE can be used to predict future breast cancer risk, broadening the clinical utility of this marker.

When we launched the BREAST Stamp Project in 2007, there was not sufficient scientific evidence to recommend supplemental MRI screening for women with dense breasts [12]. In our study, only two of 218 women (0.9 %) were recommended to have a supplemental MRI. As a result, we are unable to assess the relationship between MRI-assessed BPE and MVD in this study population. Although we did not capture MRI as part of the study protocol, increasingly, MRI is being considered as a supplemental screening tool among women with dense breasts [13], and our work may therefore be important for future studies relating breast tissue vascularity to radiologic measures of breast density.

In a cohort of approximately 2000 women who underwent a biopsy for a benign breast lesion, 24 women subsequently developed breast cancer (median time to diagnosis was not provided) [14]. For each case, one control subject who did not develop breast cancer in follow-up was randomly selected and matched by age at biopsy, year of biopsy, and follow-up time. Tissue from the initial biopsy lesion was assessed for MVD, and the authors reported a seven times higher breast cancer risk (95 % CI = 0.9–52.2) for women in the highest category of MVD compared with the lowest [14]. Other studies have evaluated MVD levels across a spectrum of breast histology in convenience samples, including benign tissue from patients undergoing reduction surgery, hyperplasia, in situ, and invasive breast cancers [15–18]. These studies have demonstrated a gradient of higher MVD with increasing lesion severity, which was also evident in our multivariable polytomous logistic regression analyses that separately considered the non-benign diagnoses.

We initially hypothesized that the combination of high MD and high MVD would be significantly associated with breast cancer compared with other combinations of these two features (e.g., high MD/low MVD). The underlying rationale for this hypothesis was two-fold: first, while higher mammographic breast density is strongly associated with breast cancer risk, many women with dense breasts do not subsequently develop breast cancer. Therefore, other biological mechanisms must work in concert with elevated density to increase breast cancer risk. Second, higher levels of BPE, a measure of vascular supply, are positively associated with breast cancer independent of fibroglandular content [5, 6]. We posited that dense breast tissue characterized by high vascularity would be metabolically “active” with a higher potential for malignant transformation compared with dense, avascular breast tissue. We indeed observed a higher odds of breast cancer associated with higher MVD within strata of high absolute density measures; however, these associations were not statistically different than the effects observed within strata of low absolute density measures.

We observed that the MVD-breast cancer association was stronger among women with greater amounts of non-dense tissue, as revealed in the analyses stratified by percent density and absolute non-dense volume. Regardless of the density measure (global or lesional), these analyses consistently demonstrated a significant positive OR for the relationship between MVD and breast cancer odds within strata of low percent density and within strata of high absolute non-dense volume. Reasons for this association are unclear. Non-dense breast tissue is primarily composed of adipose tissue, which is radiolucent and appears dark on a mammogram. In a recent meta-analysis of area density measure studies, absolute non-dense area was inversely associated with breast cancer risk among pre- and postmenopausal women, with slight attenuation after adjustment for absolute dense area, particularly among premenopausal women [19]. Conversely, some have suggested that higher amounts of breast adipose tissue are related to increased breast cancer risk [20, 21]. Adipose tissue secretes a number of factors including adipokines, pro-inflammatory molecules, chemokines, hormones, and growth factors [22] – when dysregulated, these factors have been shown to contribute to the development and progression of breast cancers [23–25]. We hypothesize that in a microenvironment characterized by dysregulated pathways, in this case angiogenesis, adipose tissue may facilitate the carcinogenic process. Although additional studies are needed, our results would suggest that MVD is a potentially important biomarker in identifying a high-risk segment of women that are traditionally perceived as having lower breast cancer risk based on their MD.

We did not detect clear relationships between MVD and the quantitative assessments of volumetric MD (global or lesional) in the overall study population or stratified by menopausal status. In accordance with our findings, several MRI reports also failed to observe an association between BPE and MD [26–28]. Our menopausal status-stratified analyses conflict with a previous study of 28 women with paired breast MRIs – one available while the woman was pre-menopausal and one following menopause – that demonstrated a decline in BPE after menopause among 61 % (17/28) of women [11]. The lack of association between MVD and MD measures in our study, coupled with the observation that the MVD-breast cancer association was not substantially attenuated when MD was included in the models, suggests that MVD and MD affect breast cancer through independent biological mechanisms. Further, apart from lesional non-dense volume, we did not observe significant differences in MD measures by pathologic diagnosis, which has been previously reported in this study population [7]. Although percent MD is a strong and established risk factor for breast cancer development, higher MD may not necessarily predict breast cancer risk among women referred for biopsy [29].

We are unaware of any study relating breast cancer risk factors to histologic markers of angiogenesis among women with benign breast disease. Our analysis revealed few associations: MVD was inversely associated with age and BMI among women without breast cancer. Interestingly, BPE appears to be hormonally regulated: premenopausal women have been reported to have higher BPE levels compared with postmenopausal women [11] whereas use of tamoxifen [30] or aromatase inhibitors [31] has been associated with declines in BPE levels in the contralateral unaffected breast. Although we did not observe a statistically significant association between MVD and menopausal status, the age range of our study population was limited, which may have affected our ability to detect an association. Furthermore, we did not observe significant relationships between MVD and other tumor characteristics, probably due to low numbers of cases.

Strengths of our study include the quantitative, reliable global density measures that have been validated with respect to breast cancer risk factors [7] and risk [32, 33] and analysis of biopsies prior to surgical intervention, which can induce granulation tissue and neovascularity. Limitations of our study include the potential for misclassification of MVD. Although robust methods for MVD assessment in breast tumors exist [34], methods for benign breast tissues have not been well validated. Therefore, we undertook a rigorous and agnostic approach to score MVD in all tissue sections, masked to diagnosis. Importantly, we did not observe large differences in MVD between randomly selected regions on the whole section slides within a woman/biopsy, nor did we note differences when the number of high-power fields reviewed per tissue section was reduced. Moreover, the high intra-rater and inter-rater agreement we observed suggests our MVD assessment method was reproducible. Like many studies of MD, our study population consists of mostly white and well-educated women, and the extent to which these results apply to the general population is unknown. Further, our study population consists of women referred for an image-guided breast biopsy – identifying biomarkers among women referred to biopsy is important and future studies in larger, more diverse populations may be warranted to determine the utility of this biomarker.

In summary, our histopathologic analysis suggests that tissue vascularity, as reflected by MVD, is associated with breast cancer independently of MD, and the effect may be stronger among women with lower breast density. Although women with low MD tend to have lower breast cancer risk, these women account for a high percentage of breast cancers overall. Our results add to prior findings suggesting that the features of benign parenchyma, assessed radiologically or histologically, may be related to breast cancer. Specifically, prior analyses have found that women with benign breast disease who have more associated terminal duct lobular units (TDLUs) experience increased breast cancer risk [35, 36], independent of MD [37], although the two are correlated [9, 38]. In future studies, it may be possible to integrate MD, MVD (assessed histologically or radiologically) with histologic assessment of TDLU involution to assess future breast cancer risk, particularly following a biopsy diagnosis of benign breast disease.

## Conclusions

Microvessel density was positively associated with breast cancer odds in our study, independent of mammographic density, an established breast cancer risk factor. Further, the association between microvessel density and higher breast cancer odds was stronger among women with lower mammographic breast density, which account for a high percentage of breast cancers overall. These results need to be replicated in larger studies, particularly in prospective studies with mammograms and breast biopsy material available years prior to the cancer diagnosis. This would provide evidence that increased vascularity is related to cancer development, rather than a finding that manifests after cancer occurs.

## Additional file

Additional file 1: Table S1. Association between microvessel density (MVD) and tumor characteristics among breast cancer cases (n = 44). (DOC 54 kb)

## Abbreviations

BI-RADS: Breast Imaging Reporting and Data System
BMI: Body Mass Index
BPE: Background Parenchymal Enhancement
BREAST: Breast Radiology Evaluation and Study of Tissues
CI: Confidence Interval
CV: Coefficient of Variation
DAB: Diaminobenzadine
ER: Estrogen Receptor
ICC: Intraclass Correlation Coefficients
MD: Mammographic Density
MRI: Magnetic Resonance Imaging
MVD: Microvessel Density
NCI: National Cancer Institute
OR: Odds Ratio
PR: Progesterone Receptor
SXA: Single X-ray Absorptiometry
TDLU: Terminal Duct Lobular Unit
