# Supplementary material for: Relationships between mammographic density, tissue microvessel density, and breast biopsy diagnosis
Source: Breast Cancer Res. 2016 Aug 23;18(1):88. doi: 10.1186/s13058-016-0746-9 (PMC4995674; doi:10.1186/s13058-016-0746-9)
Supplement: Additional file 1: Table S1. — Association between microvessel density (MVD) and tumor characteristics among breast cancer cases (n = 44). (DOC 54 kb) [file 13058_2016_746_MOESM1_ESM.doc]

| Additional file 1: Table S1. Association between microvessel density (MVD)^1^ and tumor characteristics among breast cancer cases (n=44) | | | |
| --- | --- | --- | --- |
| Characteristics | n | median (range) | p^2^ |
| **Grade**^3^ |  |  |  |
| DCIS |  |  | 0.10 |
| I | 3 | 1.5 (1.3-1.5) |  |
| II | 14 | 1.9 (1.0-4.6) |  |
| III | 11 | 2.8 (1.3-4.5) |  |
| Invasive cancer |  |  | 0.61 |
| I | 2 | 2.9 (2.6-3.2) |  |
| II | 4 | 4.2 (1.8-5.0) |  |
| III | 2 | 4.0 (3.0-5.0) |  |
| **Lesion size** |  |  | 0.45 |
| < 1 cm | 19 | 2.3 (1.0-4.3) |  |
| ≥ 1 cm | 24 | 3.1 (1.3-5.0) |  |
| **Histologic type** |  |  | 0.61 |
| Ductal | 34 | 2.5 (1.0-5.0) |  |
| Lobular | 8 | 2.3 (1.3-3.4) |  |
| Other | 2 | 3.2 (2.6-3.8) |  |
| **ER status** |  |  | 0.08 |
| Negative | 9 | 3.4 (1.5-5.0) |  |
| Positive | 35 | 2.1 (1.0-5.0) |  |
| **PR status** |  |  | 0.28 |
| Negative | 11 | 2.8 (1.5-5.0) |  |
| Positive | 33 | 2.1 (1.0-5.0) |  |
| 1 Average MVD within a woman was standardized by one standard deviation  2 Kruskal-Wallis p-value  3 Grade was not assessed among women with lobular carcinoma *in situ* (n=8) | | | |
